# Early Microcirculatory Hemodynamic Changes Are Correlated With Functional Outcomes at Discharge in Patients With Aneurysmal SAH

**DOI:** 10.3389/fneur.2021.793411

**Published:** 2022-01-20

**Authors:** Lili Wen, Longjiang Zhou, Qi Wu, Xiaoyu Tang, Jiajia Ge, Xiaoming Zhou, Xin Zhang

**Affiliations:** ^1^Department of Neurosurgery, Jinling Hospital, Jinling School of Clinical Medicine, Nanjing Medical University, Nanjing, China; ^2^Department of Neurosurgery, Jinling Hospital, Nanjing University School of Medicine, Nanjing, China; ^3^Advanced Therapies, Siemens Healthineers Ltd., Shanghai, China

**Keywords:** aneurysmal subarachnoid hemorrhage, color-coding, digital subtraction angiography, hemodynamic, outcome

## Abstract

**Purpose:**

The technique of color-coding blood flow analysis was used to explore the correlation between the microcirculatory hemodynamic changes on digital subtraction angiography (DSA) images in patients with aneurysmal subarachnoid hemorrhage (SAH) at the early stage and functional outcomes at discharge.

**Methods:**

Data of 119 patients who underwent DSA examination due to SAH were retrospectively analyzed. The following hemodynamic parameters of the four region of interests (ROIs) [an ophthalmic segment of the internal carotid artery (ICA), frontal and parietal lobe, and superior sagittal sinus] were analyzed: the time-to-peak (TTP), the area under the curve (AUC), the full width at half maximum (FWHM), mean transit time (MTT), and circulation time. Multifactor regression analysis was performed to explore the correlation between the hemodynamic parameters and functional outcomes in patients at discharge.

**Results:**

Of 119 patients with SAH, good and poor outcomes were found in 83 (69.7%) and 36 (30.3%) patients, respectively. The hemodynamic parameters including the FWHM, relative TTP (rTTP), and circulation time were significantly correlated with the Hunt–Hess grade (*p* < 0.005, *p* = 0.03, and *p* < 0.005) and the World Federation of Neurological Societies Scale grade (*p* < 0.005, *p* = 0.02, and *p* = 0.01). The FWHM was significantly prolonged with the increase of modified Fisher grade (*p* = 0.02). The multifactor analysis showed that the FWHM [odds ratio (OR) 17.56, 95% CI: 1.13–272.03, *p* = 0.04] was an independent risk factor predicting the functional outcomes in patients at discharge.

**Conclusion:**

The technique of color-coding blood flow analysis could be suitable for the qualified evaluation of disease conditions at an early stage of SAH as well as the prediction of outcomes.

## Introduction

Aneurysmal subarachnoid hemorrhage (SAH) is a critical disease with high mortality and disability rates. Increasing fundamental and clinical research has suggested that early brain injury is the most critical cause of delayed neurological dysfunction, mortality and disability in patients (1–6). Early brain injury involves a series of microcirculation dysfunctions that occur within 72 h after SAH, which is manifested as microcirculation spasm, microthrombi formation, and blood-brain barrier disruption (1, 7, 8).

Several investigators have already used imaging techniques to explore the early microcirculatory changes in patients with SAH, such as using CT perfusion (CTP) to monitor early brain injury (2, 9, 10) and using MR perfusion to investigate perfusion of cerebral tissues (11). Previous studies showed that the decrease of cerebral blood flow (CBF) was associated with the increase of mean transit time (MTT), delayed cerebral ischemia, and poor prognoses (12–15). Yet, all these methods require patients to stay in specific examination rooms, which involve increased waiting time and irradiation dosages. Therefore, such methods may not be applicable for patients with SAH requiring emergent surgeries or with critical disease conditions.

The 2-dimensional (2D) data of digital subtraction angiography (DSA)-based color-coding blood flow analysis that has been extensively used over the recent years provided another alternative for the evaluation of blood flow changes in microcirculation (8, 16–19). As this technique can be simultaneously completed in one-stop in the DSA room with surgery, patients are exempted from additional waiting time, irradiation dosages, and injuries (20). In this study, we aimed to use the color-coding blood flow analysis technique to investigate the early microcirculatory hemodynamic changes following SAH and explore the correlation between microcirculatory hemodynamic changes and clinical manifestations and functional outcomes of patients at discharge, respectively. We hypothesize that significant changes of hemodynamic parameters given by the quantitative blood flow analysis could be beneficial in SAH early phase evaluation and outcome prediction.

## Materials and Methods

### Patients

This study was approved by the Institutional Research Ethics Committee of the Jinling Hospital. Written informed consent was obtained from a legally authorized representative of all the patients. In total, 467 patients diagnosed with aneurysmal SAH who received endovascular embolization in the Jinling Hospital between January 1, 2016, and December 31, 2017, were retrospectively analyzed. The exclusion criteria were following: (1) the time interval exceeded 48 h from hemorrhage to surgery; (2) DSA or CT angiography (CTA) suggested the presence of cerebrovascular malformation, moyamoya disease, moderate or higher degree cerebral artery stenosis, or other cerebrovascular diseases; and (3) with contraindications to DSA examination, such as pregnancy, renal impairment, or allergic to the contrast agent. Clinical records related to functional outcomes including age, gender, the Hunt–Hess grade, the World Federation of Neurological Societies Scale (WFNSS), the modified Fisher (mFisher) grade, and location of aneurysm were collected for analysis.

### Methods

All patients received full-brain DSA examination (Artis Zee Biplane DSA machine; Siemens Healthineers, Erlangen, Germany) through the transfemoral artery approach. Bilateral internal carotid arteries and bilateral vertebral arteries were examined for all patients to acquire standard anteroposterior and lateral DSA sequence images. Iodixanol (Visipaque, 320 mg I/ml) was used as the contrast agent. The parameters of the contrast agent were as follows: a total dosage of 7 ml and injection velocity of 5 ml/s for the internal carotid artery, a total dosage of 5 ml and injection velocity of 3 ml/s for the external carotid artery, a total dose of 6 ml and injection velocity of 4 ml/s for the vertebral artery, and acquisition rate of 8 frames/s. The images were acquired from the early arterial phase to the complete display of the sigmoid sinus.

**Figure 1:**
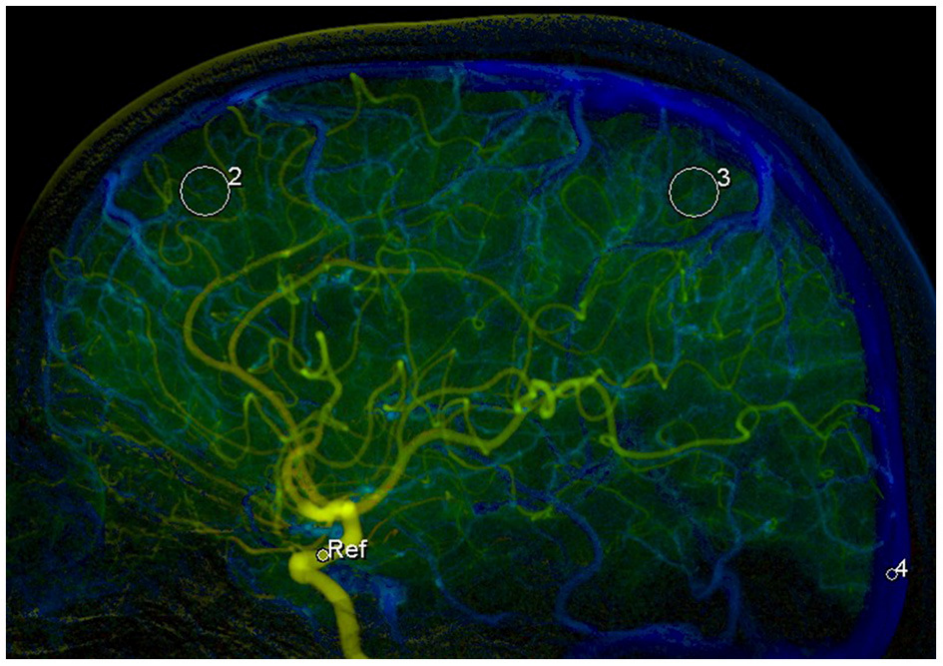
Selection of the 4 regions of interests (ROIs) in the lateral phase of colored blood flow image: 1, the ROI with areas of 2.4 mm^2^ at the ophthalmic segment of the internal carotid artery (ICA); 2, the ROI with areas of 42.6 mm^2^ at frontal lobe; 3, the ROI with areas of 42.6 mm^2^ at parietal lobe; and 4, the ROI with areas of 2.4 mm^2^ at superior sagittal sinus at the 2 cm above the confluence of the sinus.

**Figure 2:**
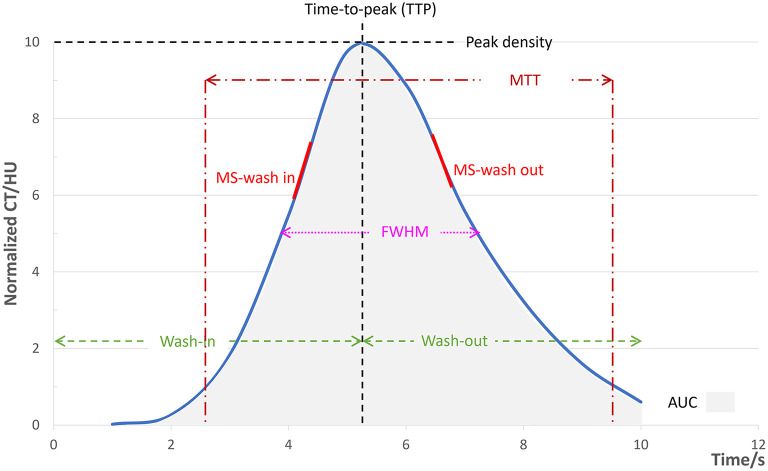
Illustration diagram of each parameter. The simulated TDC according to the time-contrast agent intensity curve by the Matlab software and the hemodynamic parameters calculated from the TDC. TDC, time-density curve; FWHM, full width at half maximum. MTT, mean transit time; MS, maximal slope; AUC, area under curve.

The original 2D DSA data acquired during the examinations were transferred to the image postprocessing workstation (Syngo XWP, Siemens Healthineers, Erlangen, Germany), and the color-coding analysis software (Syngo iFlow; Siemens Healthineers, Erlangen, Germany) was used for the color-coding of 2D DSA data. The hue, saturation, and value of each pixel were transformed to a specific color to acquire a colored blood flow image of the whole processes of the flow of contrast agent in blood vessels. The area of the ophthalmic segment of the internal carotid artery, frontal lobe, parietal lobe, and the superior sagittal sinus at the 2 cm above the confluence of the sinus was selected as the region of interest (ROI) in the lateral phase of colored blood flow images (Figure 1; white circles indicated the ROI). The data of time-contrast agent intensity in the ROI were then acquired. The selection and standardization of the ROI area follow the rules with respect to the calibers of the selected vessels or avoid overlapping with anatomical structure and inhomogeneous areas (21). Details are as follows: 2.4 mm^2^ for the ROI at the ophthalmic segment of the internal carotid artery and sigmoid sinus and 42.6 mm^2^ for the ROI at the frontal lobe and parietal lobe. When choosing the ROI at the frontal lobe and parietal lobe, the areas with traveling arteries or veins were avoided. The data of time-contrast agent intensity were simulated to acquire the time-density curve (TDC) by the Matlab software. The following hemodynamic parameters were calculated by the TDC: (1) the time-to-peak (TTP), which reflects the time of contrast agent to reach the peak in the ROI; (2) peak density, which reflects the peak value of the TDC and indicates the highest grayscale value of contrast agent intensity in the ROI; the peak is correlated with blood flow volume when the parameters of imaging are not changed; (3) the area under curve (AUC), which reflects the blood volume in the ROI; (4) the full width at half maximum (FWHM), which reflects the time required for contrast agent intensity to reduce from the highest value to half in the ROI and indirectly reflects the time of blood flow through the tissue; (5) maximal slope (MS) of wash-in and wash-out (MS wash-in and MS washout), which reflects the velocity of inflow and outflow blood; (6) mean transit time (MTT), namely the time from 10 to 90% on the horizontal axis of the TDC, reflecting 80% of the time from inflow to complete outflow of contrast agent in the ROI; and (7) circulation time (CirT), namely the TTP difference from the ROI at the ophthalmic segment of the internal carotid artery to sigmoid sinus, which reflects the total time of cerebral circulation from the inflow in the artery to outflow in the vein (Figure 2).

The microvascular TTP was reflected by the relative TTP (rTTP), which was calculated by the equation: rTTP = [(TTP_frontal lobe ROI_ – TTP_ophthalmic segment ROI_) + (TTP_parietal lobe ROI_ – TTP_ophthalmic segment ROI_)]/2. The microvascular TT was reflected by the relative MTT (rMTT), which was calculated using the following equation: rMTT = [(TTP_superior sagittal sinus ROI_ – TTP_frontal lobe ROI_) + (TTP_superior sagittal sinus ROI_ – TTP_parietal lobe ROI_)]/2. MS wash-in, MS washout, the AUC, and peak density were all reflected by the ratio of the frontal lobe and parietal lobe ROI to the reference, ophthalmic segment of internal carotid artery ROI. The CirT was calculated by the equation: CirT = (TTP_superior sagittal sinus ROI_ – TTP_ophthalmic segment ROI_).

### Clinical Outcomes

The modified Rankin Scale (mRS) of patients at discharge was used to reflect the neurological functions. The mRS of 0–2 points indicated good outcomes and 3–6 points indicated poor outcomes.

### Statistical Analysis

Continuous variables are presented as mean with SD and/or range when appropriate. Comparisons between groups were performed with ANOVA for continuous parameters and the chi-squared test for categorical parameters. Significant univariate factors with a *p*-value ≤ 0.1 were entered into the multivariate logistic regression. Odds ratios and associated 95% CIs are reported for regression analysis. Statistical analysis was performed using IBM SPSS Statistics software version 19.0 (IBM Corporation, Armonk, New York, USA). *P*-value < 0.05 was considered as statistically significant.

## Results

In total, 119 patients, 45 men (37.8%) and 74 women (62.2%) with average age of 55.92 ± 10.41 years (33–82 years), were included in this study. The outcomes were good for 83 patients (69.7%) at discharge and were poor for 36 patients (30.3%) (Table 1).

**Table 1:** Demographics and clinical features of aneurysmal patients with SAH.

**Characteristics**	**All patients (*N =* 119)**	**Good outcome (*n =* 83)**	**Poor outcome (*n =* 36)**	***P* Value**
Gender, *n* (%)				0.84
Male	45 (37.8%)	32 (38.6)	13 (36.1%)	
Female	74 (62.2%)	51 (61.4%)	23 (63.9%)	
Age (years), mean ± SD	55.92 ± 10.41	53.29 ± 9.49	62.00 ± 9.99	<0.005
Hunt-hess, *n* (%)				<0.005
I	2 (1.7%)	2 (2.4%)	0 (0%)	
II	66 (55.5%)	63 (75.9%)	3 (8.3%)	
III	25 (21.0%)	13 (15.7%)	12 (33.3%)	
IV	21 (17.6%)	4 (4.8%)	17 (47.2%)	
V	5 (4.2%)	1 (1.2%)	4 (11.1%)	
WFNSS, *n* (%)				<0.005
I	43 (36.1%)	41 (49.4%)	2 (5.6%)	
II	36 (30.3%)	30 (36.1%)	6 (16.7%)	
III	11 (9.2%)	7 (8.4%)	4 (11.1%)	
IV	16 (13.4%)	1 (1.2%)	15 (41.7%)	
V	13 (10.9%)	4(4.8%)	9 (25.0%)	
mFisher, *n* (%)				<0.005
0	2 (1.7%)	2 (2.4%)	0 (0%)	
I	3 (2.5%)	2 (2.4%)	1 (2.8%)	
II	30 (25.2%)	30 (36.1%)	0 (0%)	
III	45 (37.8%)	32 (38.6%)	13 (36.1%)	
IV	39 (32.8%)	17 (20.5%)	22 (61.1%)	
Aneurysm site, *n* (%)				0.65
ICA	48 (40.3%)	36 (43.4%)	12 (33.3%)	
MCA	13 (10.9%)	8 (9.6%)	5 (13.9%)	
Anterior cerebral artery	1 (0.8%)	0 (0%)	1 (2.8%)	
Anterior communicating artery	39 (32.8%)	27 (32.5%)	12 (33.3%)	
Basilar artery	8 (6.7%)	6 (7.2%)	2 (5.6%)	
Veterbral artery	6 (5.0%)	4 (4.8%)	2 (5.6%)	

*WFNSS, World Federation of Neurological Societies Scale; mFisher, modified Fisher; SAH, subarachnoid hemorrhage*.

The comparison of the ROI at bilateral frontal lobes showed that the hemodynamic parameters, including MS wash-in, MS washout, FWHM, rMTT, rTTP, AUC, and peak density, were not significantly different (*p* > 0.05). The ROI at bilateral parietal lobes also did not significantly differ (*p* > 0.05). In contrast, compared with the frontal lobe ROI, the parietal lobe ROI showed significant difference in MS washout (*p* = 0.03), FWHM (*p* = 0.01), rMTT (*p* < 0.005), peak density (*p* = 0.01), and a significantly prolonged rTTP (*p* < 0.005), while the MS wash-in and the AUC were not significantly different (*p* > 0.05) (Table 2). The FWHM (*p* < 0.005), rTTP (*p* = 0.03), and CirT (*p* < 0.005) were significantly different among the different Hunt–Hess grades. The FWHM (*p* < 0.005), rTTP (*p* = 0.02), and CirT (*p* = 0.01) also significantly differed among the different WFNSS grades. However, only the FWHM (*p* = 0.02), but not other parameters, was significantly different among the different mFisher grades (Table 2).

**Table 2:** Comparison of hemodynamic parameters derived from the time-density curve with clinical features.

	**MS wash-in**	**MS wash-out**	**FWHM**	**rMTT**	**rTTP**	**Area under curve**	**Peak density**	**CirT**
ROI								
Frontal lobe	1.36 ± 0.75	1.31 ± 0.57	2.48 ± 0.62	3.34 ± 1.11	2.81 ± 0.73	0.29 ± 0.14	0.18 ± 0.06	-
Parietal lobe	1.46 ± 0.77	1.40 ± 0.71	2.57 ± 0.66	2.88 ± 1.07	3.28 ± 0.86	0.31 ± 0.15	0.19 ± 0.08	-
*P* Value	0.05	0.03	0.01	<0.005	<0.005	0.06	0.01	-
Hunt-Hess								
I–II	1.30 ± 0.59	1.36 ± 0.62	2.22 ± 0.54	3.05 ± 1.03	2.90 ± 0.62	0.29 ± 0.13	0.19 ± 0.07	5.77 ± 1.08
III	1.50 ± 0.84	1.21 ± 0.50	2.60 ± 0.70	3.02 ± 1.08	3.14 ± 0.76	0.32 ± 0.14	0.18 ± 0.06	5.93 ± 1.52
IV–V	1.61 ± 0.82	1.48 ± 0.61	3.00 ± 0.52	3.38 ± 1.08	3.33 ± 0.94	0.33 ± 0.14	0.19 ± 0.06	7.18 ± 1.49
*P* Value	0.11	0.25	<0.005	0.36	0.03	0.36	0.75	<0.005
WFNSS								
I–II	1.36 ± 0.63	1.36 ± 0.64	2.34 ± 0.64	3.18 ± 1.10	2.92 ± 0.64	0.30 ± 0.14	0.19 ± 0.07	5.91 ± 1.27
III	1.25 ± 0.51	1.23 ± 0.42	2.04 ± 0.38	2.62 ± 0.66	3.15 ± 0.51	0.26 ± 0.14	0.16 ± 0.06	5.77 ± 0.92
IV–V	1.59 ± 0.93	1.38 ± 0.54	2.98 ± 0.53	3.33 ± 0.99	3.35 ± 0.97	0.32 ± 0.12	0.19 ± 0.07	6.78 ± 1.61
*P* Value	0.25	0.77	<0.005	0.17	0.02	0.51	0.46	0.01
mFisher								
0	1.74 ± 0.40	1.48 ± 0.28	1.63 ± 0.33	3.27 ± 0.66	2.33 ± 0.28	0.34 ± 0.02	0.25 ± 0.01	5.60 ± 0.38
I	0.94 ± 0.20	1.41 ± 0.62	2.20 ± 0.71	3.51 ± 1.36	3.60 ± 0.13	0.20 ± 0.02	0.13 ± 0.05	7.11 ± 1.47
II	1.52 ± 0.74	1.52 ± 0.74	2.21 ± 0.57	2.96 ± 0.85	2.79 ± 0.63	0.28 ± 0.12	0.19 ± 0.07	5.86 ± 1.22
III	1.31 ± 0.69	1.26 ± 0.56	2.55 ± 0.68	3.21 ± 1.15	3.13 ± 0.80	0.31 ± 0.14	0.18 ± 0.07	6.05 ± 1.39
IV	1.45 ± 0.73	1.33 ± 0.52	2.64 ± 0.64	3.08 ± 1.10	3.14 ± 0.75	0.32 ± 0.14	0.20 ± 0.06	6.34 ± 1.50
*P* Value	0.49	0.48	0.02	0.84	0.08	0.46	0.29	0.43

*WFNSS, World Federation of Neurological Societies Scale; mFisher, modified Fisher; FWHM, full width at half maximum; rTTP, relative time-to-peak; rMTT, relative mean transit time; CirT, circulation time*.

The parameters including the FWHM (*p* < 0.005), rTTP (*p* = 0.01), and CirT (*p* < 0.005) resulted as significantly different between patients with good and poor outcomes. The FWHM, rTTP, and CirT were shorter in patients with good outcomes (Table 3).

**Table 3:** The multivariate logistic regression analysis of factors associated with outcome at discharge.

	**Good outcome (mean ±sd)**	**Poor outcome (mean ±sd)**	**ANOVA**	**Multi-variate regression**		
			***P* value**	**OR**	**95% CI**	***P* value**
FWHM	2.31 ± 0.57	2.78 ± 0.64	<0.005	17.56	1.13–272.03	0.04
rTTP	2.93 ± 0.68	3.31 ± 0.83	0.01	12.08	0.61–240.98	0.10
CirT	5.86 ± 1.22	6.74 ± 1.57	<0.005	0.35	0.05–2.60	0.31
rMTT	3.00 ± 1.01	3.38 ± 1.11	0.09	2.03	0.37–11.28	0.42
MS wash-in	1.35 ± 0.62	1.53 ± 0.87	0.21			
MS wash-out	1.41 ± 0.63	1.22 ± 0.48	0.10	0.11	0.00–1.82	0.12
Area under curve	0.29 ± 0.13	0.32 ± 0.15	0.28			
Peak density	0.19 ± 0.06	0.18 ± 0.07	0.73			
Age			<0.005	1.39	1.12–1.72	<0.005
Hunt-Hess			<0.005	152.98	2.19–10701.52	0.02
WFNSS			<0.005	0.80	0.22–2.94	0.74
mFisher			<0.005	19.44	0.92–411.47	0.06

*FWHM, full width at half maximum; rTTP, relative time-to-peak; CirT, circulation time; rMTT, relative mean transit time; WFNSS, World Federation of Neurological Societies Scale; mFisher, modified Fisher*.

The multivariate logistic regression results showed that after adjusting the baseline errors of age, the Hunt–Hess grade, the WFNSS grade, and the mFisher grade, only the FWHM resulted as the independent risk factor predicting the outcomes in patients [odds ratio (OR) 17.56, 95% CI: 1.13–272.03, *p* = 0.04] (Table 3).

## Discussion

In this study, we have demonstrated the correlation between early DSA imaging-based microcirculatory hemodynamic changes and functional outcomes in patients with SAH at discharge, and the correlation between the severity of disease and microcirculatory hemodynamic changes. Compared to patients with poor recovery, the ones with good recovery had significantly shorter FWHM, rTTP, and CirT on the time-density curve of rheogram. These findings suggested that for patients who have recovered well, microcirculation resistance and damage may be lower and blood flow damage may be mild. In addition, the FWHM was also significantly related to the initial clinical manifestations of patients. It was found that the higher Hunt-Hess grade and WFNSS grade, the longer the FWHM; the higher mFisher grade, the longer the FWHM. These findings indicated that the FWHM calculated from the color-coding flow analysis based on 2D-DSA has the potential to characterize early microcirculatory changes in acute patients with SAH and predict their clinical outcomes during the initial surgery procedure, which could save a lot of time and suggest appropriate postoperation care delivery.

As aforementioned, early brain injury plays a critical role in brain dysfunction after aneurysmal SAH (1–6). Although various scoring systems have been developed, such as the Hunt–Hess, the WFNSS, and the mFisher, to help evaluate the clinical severity, recommend appropriate treatments and predict prognosis, they may be difficult to use, especially for junior clinicians and those lacking objectivity. Therefore, with the changes in the diagnosis and treatment of aneurysmal SAH, it is still not clear which proposed system is the best (22). CTP and MR perfusion (MRP) are major quantitative approaches to characterizing early cerebral perfusion and microcirculation after aneurysmal SAH, but may not be applicable to all emergency patients due to time urgency. DSA is a routine technique during the procedure for visualizing the changes in the diameter of the cerebral artery lumen and the migration information of the contrast agent in the cerebral blood vessels and tissues. However, blood flow assessment under DSA can be affected by factors such as the experience of the operators, the resolution of the angiography system, and the working angle of angiography, which may result in a lack of objectivity and accuracy. The technique of color-coding blood flow analysis converts the cerebral blood flow information into quantitative TDC without additional contrast agent or fluoroscopy, the data of which could be further analyzed to define hemodynamic parameters, such as the TTP, peak density, MTT, AUC, and FWHM, therefore allowing for a more quantifiable and immediate understanding of the microcirculatory hemodynamic changes following SAH without patient transfer (8, 16–19). A number of studies have demonstrated that the TDC is closely related to the severity of brain injury after aneurysmal SAH. Ivanov et al. demonstrated that TTs were significantly correlated with the Hunt–Hess grade (8) and the value of TT_100−10_ had more prominent significance than the values of TT_0−100_ and TT_25−100_, which was in agreement with our findings. Golitz et al. showed that patients with delayed cerebral ischemia manifested as a substantial extension of microcirculatory MTT, as well as an extension of CirT and the cerebral cortex TTP, although these extensions were not statistically significant (23). On the other hand, the study performed by Burkhardt et al. indicated that MTT was a significant indicator in patients with delayed cerebral ischemia, while CirT showed no statistically significant difference (24). However, to date, there has been no research report using the FWHM to study microcirculation hemodynamic changes after SAH.

FWHM of the TDC reflects the width of the curve and the time of blood inflow and outflow in the ROI indirectly (25). According to Ivanov et al., the value of the FWHM was similar to TT_100−10_, TT_0−100_, and TT_25−100_ (8). Compared to TT, the FWHM has the advantage that even if the entire processes of contrast agent inflow and outflow cannot be obtained when acquiring the DSA data, the FWHM indicators can still be obtained through the TDC analysis. In this study, a complete TDC could not be acquired in 31 patients (26.1%) because the ray collection time is not enough and, thus, the MTT of some selected ROIs, namely, the TT_10−90−10_ value, was not available for the analysis. Perhaps, this was the most common factor that prevents the acquisition of the intact TDC clinically. However, when the difference in the TTP between the ROI and superior sagittal sinus is used instead of MTT, that is, rMTT in this study, this value may not reflect the TT of blood flow in the microcirculation (23). Our results also indicated that rMTT was less sensitive than the FWHM in predicting clinical severity and prognosis. Therefore, the FWHM can be used as an alternative to circulating MTT to more accurately reflect changes in local microcirculatory blood flow, which is demonstrated by our findings that the FWHM could reflect the severity of clinical manifestations of patients and the mFisher grade. In addition, the FWHM was also an independent factor predicting the clinical outcomes in patients. More substantial extension of the FWHM could indicate more severe microcirculatory injuries, lower local blood flow reduction, and slower velocity of microcirculation. The FWHM was also significantly extended in cases with higher clinical grades (the Hunt–Hess grade and the WFNSS grade) and the mFisher grades. Therefore, a prolonged FWHM could increase the risks of poor outcomes in patients and also reflect the severity of microcirculatory injuries.

Although the single-factor analysis showed that the parameters including rMTT, rTTP, and CirT were correlated with the neurological functions of patients at discharge, the multifactor regression analysis failed to identify the predictive values of these parameters after the baseline characteristics were adjusted. Consistent with our findings, Golitz et al. also showed that the MTT of microcirculation was substantially extended, and CirT and the cerebral cortex TTP were also increased in the early stage of delayed brain hemorrhage; yet, the differences were not statistically significant (23). In contrast, the study performed by Burkhardt et al. showed that MTT was statistically significant (24). The differences in the results could be associated with the fact that the technique of color-coding blood flow analysis was essentially the expanded application of 2D imaging and the images acquired were still 2D images and, thus, the concentration of contrast agent, starting and ending point of contrast agent, the direction of X-ray, overlap of blood vessels, the status of blood flow, duration of DSA, and shifting of patients could all influence the accuracy and reliability of the parameters of color-coding images (16, 24–27). Therefore, more studies are needed to further investigate the accuracy and reliability of microcirculatory hemodynamic parameters acquired by color-coding blood flow analysis.

There are several limitations to this study: (1) this was a preliminary study with a relatively small sample size and (2) for several patients, data collection was performed before the contrast agent completely passed the sigmoid sinus, which could result in missing data and consequently bias the findings.

The findings of this study demonstrated that the FWHM, rTTP, and CirT were significantly prolonged in patients with high-grade SAH (the Hunt–Hess grade and the WFNSS grade IV–V). Patients with higher mFisher grades also showed significantly prolonged FWHM. These findings suggested that the microcirculation is already damaged in the early phase of SAH, and the severity of microcirculation damages could be more severe in patients with more severe disease conditions. The TDC parameters, including the FWHM, rTTP, and CirT, could relatively reflect the severities of the injuries.

## Conclusion

We have demonstrated that by using a DSA-based color-coding blood flow analysis software, microcirculatory hemodynamic changes in the early stage of aneurysmal SAH were able to be detected immediately during the surgery procedure. Significant prolonged FWHM, rTTP, and CirT were found in patients with high-grade SAH (the Hunt–Hess grade and the WFNSS grade IV–V). The change of the FWHM was significantly related to the initial disease severity of the patient and was an independent risk factor for predicting the short-term prognosis of the patient. Color-coding blood flow analysis technique could be applied to the qualified evaluation and prognosis prediction of SAH at an early phase.

## Data Availability Statement

The original contributions presented in the study are included in the article/supplementary material, further inquiries can be directed to the corresponding author.

## Ethics Statement

The studies involving human participants were reviewed and approved by the Institutional Research Ethics Committee of Nanjing Jinling Hospital. The patients/participants provided their written informed consent to participate in this study.

## Author Contributions

LW and XZha contributed to the study concept and study design. LW, QW, and XZho contributed to the data acquisition. LW and JG contributed to the quality control of data and algorithms and editing of the manuscript. JG, LZ, and XT contributed to the statistical analysis, interpretation, and preparation of the manuscript. XZha contributed to the review of the manuscript. All authors contributed to the article and approved the submitted version.

## Funding

This study was supported by the Jiangsu Planned Projects for postdoctoral Research Funds (No. 2019k281), the Jiangsu Natural Science Foundation (No. BK20191231), and the Jiangsu Natural Science Foundation (No. SBK2019022915).

## Conflict of Interest

Author JG was employed by company Siemens Healthineers Ltd. The remaining authors declare that the research was conducted in the absence of any commercial or financial relationships that could be construed as a potential conflict of interest.

## Publisher's Note

All claims expressed in this article are solely those of the authors and do not necessarily represent those of their affiliated organizations, or those of the publisher, the editors and the reviewers. Any product that may be evaluated in this article, or claim that may be made by its manufacturer, is not guaranteed or endorsed by the publisher.
